# Should the Dental Practitioner Concern Himself with the Chemical Examination of Saliva or Urine?

**Published:** 1905-01

**Authors:** H. Carleton Smith

**Affiliations:** Natick, Mass.


### SHOULD THE DENTAL PRACTITIONER CONCERN HIMSELF WITH THE CHEMICAL EXAMINATION OF SALIVA OR URINE?[1]

BY H. CARLETON SMITH, PH.D., NATICK, MASS.[2]

Unless I am mistaken, and I hope I am, an unqualified affirmative answer to the question presented for our consideration to-night would meet with considerable objection.

The reasons given might be, first, that such examination is not practicable, or, second, the information so gained may be better obtained in some other way; third, such information has slight bearing upon the problems and conditions constantly presenting themselves to a busy dentist.

In regard to these objections, the first one of non-practicability is valid only to a limited extent, and the difficulty here is not so much in making the analysis as it is in getting the samples to analyze. Under the existing social and professional usages we can imagine circumstances when it would be a little hard for a

---

[1] Read before the American Academy of Dental Science, October 5, 1904.

[2] Instructor in Chemistry, Harvard School.

dentist to secure samples that the physician asks for almost from habit. But this would pass.

The second objection, of better ways to the same end, has but little of truth behind it, and that little is constantly decreasing as we learn better and more complete methods of chemical investigation.

The third objection is a reasonable one, and merits our consideration; we might take it for our topic and restate the question for discussion: "Is a knowledge of the relationship between the chemical constituents of urine and saliva and the diseases of the oral cavity of practical value?"

I am a firm believer in the soundness of deductions from analogies. I believe in the first part of the theory of evolution because the last part is before our eyes, and, because the threefold nature of the material world and the threefold nature of man are so plainly manifest I claim a foundation for, and a right to believe in, the threefold nature of some other things concerning which we have less positive material evidence, and so, from among the lesser things of this class, I would call attention to the threefold nature of dental disease, or, better, of the three causes of dental disease which are not nearly so independent as it may at first seem.

Disease may be due, first, to an influence wholly external to the tissues involved, and which we may regard as a mechanical or, in a restricted sense, a chemical cause, as mechanical abrasions or chemical erosion. This is perhaps the least important, and appears as a primary factor in the smallest number of cases; second, to the growth of specific foreign organisms, bacteria, fungi, etc., which is also chemical and may be both external and internal as regards the tissue involved; and third, to malnutrition of the part which is a factor, perhaps in some instances secondary, but nevertheless a factor in *every* disease the nature of which is in any degree chronic.

Again, let us neglect the first two and for a little consider the third part of the proposition before us.

Malnutrition is not necessarily *lack* of nutrition, but often the wrong kind of nutrition; that is, the blood may be abundantly supplied with all the essentials to a vigorous maintenance of its physiological integrity, but the proportion of acid-forming elements may be in excess of the normal, the chemical equilibrium is disturbed and conditions are produced favorable to the development of dental disease,—pyorrhœa, for instance.

Again, there may be a very evident lack of some particular tissue constituent, such as the phosphates, and for some reason the phosphates taken into the system with a view to the correction of such lack pass into the fæces, with but very slight absorption. A case of precisely this kind came to my knowledge during last winter.

Now, is a knowledge of conditions such as these of value to the dentist? It seems to me so, especially if he realizes that his profession is not a trade, but a specialized department of medicine, as is surgery, or bacteriology, and in no way inferior to them or to medicine itself in its opportunities for profound scientific investigation or in its demand for an ability to draw *correct* conclusions from observed phenomena.

If there are diseases of the oral cavity which a dentist is expected to treat, and which may have their etiology in some systemic derangement not easily apparent, then in such cases it becomes the business of the practitioner to inform himself as thoroughly as possible as to the exact conditions existing in the patient's system, and, more than this, as to the tendencies or "diatheses" which will probably produce conditions favorable or detrimental to the future development of such diseases. In other words, if diseases of systemic nature or origin are to receive the attention of the dentist, he should obtain all possible light on his case, and the analysis of the urine throws considerable on diseases of this class. The examination of the urine, however, may be of little or no value if made with a view to the diagnosis of renal disease, and it is usually from this view-point, and in too many instances from this alone, that the physician makes his analysis. If, on the other hand, the examination of urine is made to obtain the greatest possible amount of information regarding vital processes and the body metabolism, it should be of considerable value, at least in any case where there is a possibility of constitutional disturbance.

Urine analysis may be made in different ways to tell different kinds of stories. Not different stories with contrary indication, but different kinds of stories, supplementary to each other. The dentist is not benefited in his work by a knowledge of just the variety of acute nephritis from which his patient may happen to be suffering; in fact, the patient with acute nephritis or acute anything else, unless it be a toothache, usually postpones his visit to the dentist, and acute disease (not dental) is of little importance, except for its history; but the case is different when we come to the consideration of chronic maladies. The chronic disease means impaired function and diminished vitality somewhere or somehow, and its effect on the proper nutrition of teeth and gums is worthy of consideration.

Dr. Metcalf, of New Haven, puts it well when he says that "We do know that normal function is dependent upon normal structure, and it follows that, disease being the perversion of function, and normal function depending entirely upon healthy or normal blood supply, an intimate knowledge of the chemistry of blood and nerve supply would seem indispensable."

The diseases of nutrition and faulty metabolism are receiving a constantly increasing amount of attention; but positive conclusions of a practical nature are difficult to obtain, because experimenting for long periods of time upon human subjects is impracticable and in many particulars impossible, and because the parallelism between laboratory or animal experimentation and corresponding conditions in the human organism may be very far from perfect.

We have referred to pyorrhœa, and there is no disease better suited or upon which more work has been done or may be done to demonstrate the affirmative side of the question of the evening.

I quote the following from a review in the *Dental Cosmos* of an article in the *New York Medical Journal* by Geo. F. Souwers, of Philadelphia: "The cases of pyorrhœa alveolaris which have come under the author's observation have led him to attach considerable importance to the constitutional factor, rheumatism, gout, etc., as bearing upon the etiology of this malady. Pyorrhœa alveolaris of the constitutional type should be treated systemically. If rheumatism or some other vitiated state is found to be a factor in the causation of the disease, a treatment should be instituted having in view the eradication of the constitutional taint." Analyses of urine and saliva will determine the existence of "vitiated states." While I have used this quotation because it supports my own theories on the subject, I fail to understand how the author can refer to rheumatism as a factor in the causation of pyorrhœa, or how a Boston dentist, in a paper recently read before the Northeastern Dental Association, can seriously ask the question, Why, if pyorrhœa is of constitutional origin, do not the gums show signs of some increased irritation in cases of acute rheumatism or gout?

As I understand pyorrhœa, it is a disease of itself, that is, a product of faulty conditions of some sort, and not secondary to gout or, of necessity, to any disease. If this is true, it is as proper to speak of Riggs's disease as the cause of gout as is the converse, or there is as much reason to question that gout is a uric acid disease because people suffering from acute rheumatic affection do not show indications of gout. Because the excess of uric acid in the blood is going in a given direction is, of itself, a reason why it should not at the same time go in another direction. Rheumatism and gout are seldom simultaneous manifestations of the uric acid diathesis. This may be too much of a digression. I do not care to provoke a discussion of the etiology of pyorrhœa, but I *do* want to cite a few cases, as of one of the diseases where an analysis of the urine will be of assistance to the dentist. This will be particularly true if, as I believe, there may be cases of pyorrhœa which are *not* caused by uric acid poisoning.

During the past year I have examined the urine from a number of patients suffering from pyorrhœa, and in the great majority of cases the results would justify placing uric acid among the probable causative factors of the disease. A patient of Dr. V. C. Pond showed increased uric acid in nine out of ten analyses made since June, 1902.

I have had a number of specimens from Drs. C. P. and E. C. Briggs, and of these all have shown excessive amounts of uric acid, with one exception; this happened to be among the first received, and was unmarked. Of the other samples received from the Drs. Briggs, I note particularly three marked Mrs. C. S. D., M. C. G., and Mrs. C. S., in all of which the uric acid was very high and the hyperacidity very marked.

Dr. L. M. Miner sent a sample marked J. M. M., also showing high uric acid, and excessive acidity; incidentally it may be stated that the acidity is *not* due, more than to slight extent, to the presence of uric acid. The uric acid is either combined with the alkaline bases as neutral or acid urates, or if in a free state it is very insoluble in acid urine and appears in crystalline form in the sediment.

Dr. D. W. Dickinson also sent one sample with high uric acid and high acidity; this case also contained sodium oxalate; it was marked J. B. I have noted the presence of oxalates in other samples which have shown high uric acid, but cannot say from my observations thus far how general the occurrence may be.

Samples have also been received from Dr. H. W. Hardy, marked G. I. O., and from Dr. H. E. Kahn, marked D., in which the uric acid was very high, but increase of acidity was not so strongly marked. I have mentioned the initials with which these samples were marked, hoping that some of the doctors from whom they were received would be present to-night and perhaps add a word of subsequent history of the cases. These are all supposed to be cases of pyorrhœa, and an anti-uric acid treatment has been recommended. As previously intimated, laboratory experiments along these lines are not practicable, and preponderance of evidence furnished by cases in actual practice will give the most satisfactory and I believe the most conclusive information on the subject; and along this line Dr. J. Warren Achorn contributes the following: Record of three cases where Riggs's disease was materially benefited by an anti-uric acid diet, which was given for other purposes, the benefit to teeth and gums being purely incidental.

First, a woman of forty-five, weight ninety-eight pounds, sent by Dr. Robert Upham. History of canker of the tongue, had Riggs's disease of three years' standing; had been in the habit of eating in the morning red meat and coffee with considerable sugar. For lunch meat again, beef broth, and coffee; at night, roast beef or lamb, a lot of tomatoes, and strawberries whenever available.

For this was substituted a breakfast of cereal carrying a large amount of gluten, fish boiled or broiled (not fried), eggs twice a week, cestus bread and cestus phosphated crackers, cooked fruit, and cereal coffee. Lunch: White meats, chicken, turkey or game birds; fish, crackers and milk, berries (except strawberries), white grapes, and nuts. Dinner: A more extended course of the same general sort and including soups, raw oysters, plain lobster, little-neck clams, vegetables, and to a very limited extent the red meats, tomatoes, asparagus, etc. The result of this treatment for four months was marked improvement according to her dentist, not according to the physician, for he did not follow the case, but the dentist complained that this system of diet had lost him a patient.

A second case was Mr. C. H. R., of North Adams, cured of chronic recurrent lumbago in one year's time by diet similar to above but adapted, of course, to the peculiarities of the individual, as every diet list must be. At the beginning of the treatment the patient had pyorrhœa; at its finish the gums were hard and firm.

The third case was Mr. C. A. V., of Boston. Weight two hundred and thirty-three pounds; a large eater of sweets and meat. Had Riggs's disease and a tendency to apoplexy. He was treated for obesity, and out of regard to the apoplectic propensity special care was taken to reduce the blood-pressure as far as possible, and to this end all highly nitrogenous foods were eliminated from the diet, with the result that Mr. V.'s weight was reduced to one hundred and ninety pounds and his Riggs's disease was cured.

Dr. Alexander Haig, of London, a great English authority on dietetics, says, in the *Medical Record* for October 31, 1903, that "the chief advantage of a bread and fruit diet is seen in the collæmic, or *high blood-pressure group* of *uric acid food poisonings*. Such used to be called diseases, and were reckoned under the names of headache, epilepsy, obesity," etc. From such cases as those above cited, which I have every reason to believe have been accurately stated, from the testimony of physicians and dentists, from books and articles on the subject which I have had time to read, and from my own results in examinations of urine and saliva of afflicted individuals, I have come to believe in the following summary of doctrine:

First: The great majority of cases of Riggs's disease are accompanied by conditions of hyperacidity of the stomach and by high arterial tension.

Second: That this disease (which I have used to illustrate my point simply because data concerning it was most easily available), if not due to, is usually accompanied by, and aggravated by the presence of excessive amounts of uric acid in the blood, and properly comes under the above classification made by Dr. Haig, although not specifically mentioned by him.

Third: That such conditions may be shown by analyses of the urine and saliva, and may be greatly benefited by a carefully restricted diet based upon a study of the general metabolism of the patient.

Fourth: That the more complete the knowledge of the case in all its details and complications, and the more closely the patient follows exactly the doctor's directions, when based on such intimate knowledge, the better the results.

This fourth statement may seem self-evident and unnecessary, nevertheless it is a very important and a much disregarded fact.

I believe most emphatically that the up-to-date dentist of the future will make dietetics and body metabolism an important part of his study in every case dependent upon constitutional conditions. Further, physicians are more and more turning over to the dentists their patients with any sort of mouth-disease, and where complications of a systemic nature occur, the dentist must make his investigation as thoroughly as the physician in order to treat the case as intelligently.

Now I want to spend a few moments in considering *how* this information may be obtained by the dentist.

Dr. Joseph P. Michaels, of Paris, Dr. E. C. Kirk, of Philadelphia, and many others are putting considerable time and study on the relation of salivary analysis to dental diseases, and among the methods they employ a few simple tests may be selected which are easily made and will mean more as our general knowledge of the subject broadens.

Dr. Michaels first notes the physical properties of a sample of saliva,—its color, its taste (which we omit), its appearance, consistence, and odor; also the appearance under the microscope and the micro-polariscope.

Then the chemical examination, consisting of a five-drop test for five different chemical principles. The first drop is tested for acidity or alkalinity with litmus. The second for ammonium salts with Messler's reagent. The third for sulphocyanates with ferric chloride. The fourth for chlorides with silver nitrate and potassium chromate. The fifth drop for glycogen with Gram's reagent (aqueous iodine solution with potassium iodide).

To these five I would add a sixth, made with equal ease and sometimes giving indications of importance,—that is a test for acetone. The test is made by dissolving in the drop of saliva a small crystal or fragment of potassium carbonate; then a small drop of iodine solution (Gram's) will develop the odor of iodoform if acetone is present. The iodoform separates in small yellow star-like crystals, which may be easily observed under the microscope.

The picture shown by the micro-polariscope is one of great beauty, besides being intensely interesting. A great variety of crystalline forms present themselves, but with the exception of a comparatively small number their significance has not been clearly demonstrated.

The oxalates (sodium), lactophosphate, and urates (sodium) I can show you.

Dr. Michaels says, "The presence of biliary pigments in the saliva is beyond any doubt. These substances can be seen in histological preparations of the saliva of diathetic individuals; also in pathological products of diseased blood and pus of alveolo-dental pyorrhœa. The pigments are perfectly characteristic and it depends only on the technique to discover them." Now, this is one of the things I am particularly anxious to have you teach me about. I have never seen them to know what they are. I do not reflect on the accuracy of the quotation, but I have probably not yet stumbled upon the "technique."

It is along this line of investigation I would ask your assistance. I shall be glad to examine any sample of saliva which you care to send to the Laboratory of the Harvard Dental School, and which in your opinion will show marked departure of some sort from the normal. If the saliva can be accompanied by a twenty-four sample of urine, additional light can undoubtedly be thrown upon the case. These examinations I am glad to make free of all expense throughout this month of October at least.

